# Comparison of the Effect of Two Human Milk Fortifiers on Clinical Outcomes in Premature Infants

**DOI:** 10.3390/nu6010261

**Published:** 2014-01-03

**Authors:** Melissa Thoene, Corrine Hanson, Elizabeth Lyden, Laura Dugick, Leslie Ruybal, Ann Anderson-Berry

**Affiliations:** 1 Newborn Intensive Care Unit, Nebraska Medical Center, Omaha, NE 68198, USA; E-Mails: mthoene@nebraskamed.com (M.T.); ldugick@nebraskamed.com (L.D.); 2 School of Allied Health Professionals, University of Nebraska Medical Center, 984045 Nebraska Medical Center, Omaha, NE 68198-4045, USA; E-Mail: ckhanson@unmc.edu; 3 College of Public Health, University of Nebraska Medical Center, 984375 Nebraska Medical Center, Omaha, NE 68198-4375, USA; E-Mail: elyden@unmc.edu; 4 Department of Pediatrics, University of Nebraska Medical Center, Omaha, NE 68198-2185, USA; E-Mail: leslie.ruybal@unmc.edu

**Keywords:** prematurity, human milk, fortifier, infant feeding, growth, acidosis

## Abstract

The use of human milk fortifiers (HMF) helps to meet the high nutritional requirements of the human milk-fed premature infant. Previously available powdered products have not met the protein requirements of the preterm infant population and many neonatologists add powder protein modulars to help meet protein needs. The use of powdered products is discouraged in neonatal intensive care units (NICU) due to concern for invasive infection. The use of a commercially available acidified liquid product with higher protein content was implemented to address these two concerns. During the course of this implementation, poor growth and clinically significant acidosis of infants on Acidified Liquid HMF (ALHMF) was observed. The purpose of this study was to quantify those observations by comparing infant outcomes between groups receiving the ALHMF *vs*. infants receiving powdered HMF (PHMF). A retrospective chart review compared outcomes of human milk-fed premature infants <2000 g receiving the ALHMF (*n* = 23) and the PHMF (*n* = 46). Infant growth, enteral feeding tolerance and provision, and incidence of necrotizing enterocolitis (NEC), metabolic acidosis, and diaper dermatitis were compared between the two groups. No infants were excluded from this study based on acuity. Use of ALHMF resulted in a higher incidence of metabolic acidosis (*p* = 0.002). Growth while on HMF as measured in both g/kg/day (10.59 *vs*. 15.37, *p* < 0.0001) and in g/day (23.66 *vs*. 31.27, *p* = 0.0001) was slower in the ALHMF group, on increased mean cal/kg/day (128.7 *vs*. 117.3, *p* = 0.13) with nearly twice as many infants on the ALHMF requiring increased fortification of enteral feedings beyond 24 cal/ounce to promote adequate growth (48% *vs*. 26%, *p* = 0.10). Although we were not powered to study NEC as a primary outcome, NEC was significantly increased in the ALHMF group. (13% *vs*. 0%, *p* = 0.03). Use of a LHMF in an unrestricted NICU population resulted in an increase in clinical complications within a high-acuity NICU, including metabolic acidosis and poor growth. Although further research is needed to assess outcomes among infants with a variety of clinical acuities, gestational ages, and weights to confirm these findings, based on this experience, caution is urged to avoid potential risks.

## 1. Introduction

Infants born prematurely have increased nutrient needs compared to those born at term [1,2,3]. Nutrition-related goals for premature infants aim to mimic fetal nutrient accretion and growth *in utero* [4], yet many develop extrauterine growth restriction (EUGR) [5].

Despite the availability of customized, nutrient-dense enteral formulas, the American Academy of Pediatrics strongly supports the use of human milk for premature infants [6]. However, unfortified human milk remains inadequate to meet the high nutrient requirements of premature infants [1,4,7]. Provision of unfortified human milk has subsequently been linked to suboptimal growth (development of EUGR or growth < 15 g/kg/day), reduced bone density leading to osteopenia of prematurity and a clinical diagnosis of rickets, and the secondary consequences of each [1,4].

The use of commercial human milk fortifiers (HMF) allows for a more optimal provision of essential nutrients to meet premature infant requirements [1,4,7]. Macronutrient recommendations for low birth weight premature infants vary, but consensus goal ranges suggest enteral intake of 110–120 cal/kg/day and 3.4–4.4 g protein/kg/day [1]. Protein is specifically emphasized, as early and higher provisions promote more desirable growth and clinical outcomes [8,9]. The use of HMF has been shown to be both safe and effective in improving growth and nutrition status of premature infants compared to unfortified human milk [7,10,11]. In recent years the use of HMF with additional powdered protein modular has been presented as a method of supplying the preterm infant with the recommended amount of enteral protein to provide improved linear growth and neurodevelopmental outcomes [12,13].

Human milk fortifiers have primarily been available in powder form, although the United States Food and Drug Administration discourages the use of powdered forms in the neonatal intensive care units (NICU) secondary to contamination risk [14]. They additionally advise that “alternatives to powdered forms should be chosen when possible” [14]. To comply with this recommendation and achieve improved protein intake, The Nebraska Medical Center (TNMC) NICU changed standard human milk fortification practices with a powdered product when an acidified liquid HMF (ALHMF) with improved protein delivery became available. However, in the four months following our initial use of the ALHMF, clinical observations of infants receiving the ALHMF suggested an increased feeding intolerance, increased incidence of metabolic acidosis, poor growth, and a need for higher caloric densities of enteral feedings to promote adequate growth. Due to our concern for patient outcomes, use of the ALHMF was discontinued. The purpose of this study is to objectively quantify these clinical observations by comparing outcomes of infants receiving the ALHMF to those receiving the originally-used PHMF. Our study also looked to identify potential risk factors for the development of the observed clinical complications, as previous research evaluating the ALHMF also documented changes in pH and CO_2_ when compared to a powder HMF (PHMF) [15].

## 2. Patients and Methods

### 2.1. Participants and Study Design

The institutional review board at the University of Nebraska Medical Center (Omaha, NE, USA) approved this study. Data was retrospectively collected from inpatient electronic medical records of all infants admitted to the NICU, between October 2009 and July 2011, if they met the following inclusion criteria: birth weight (BW) < 2000 g, received enteral feedings as fortified maternal breast milk during NICU stay, and remained in the NICU ≥ 14 days. Exclusion criteria included infants with congenital abnormalities or conditions that significantly inhibited growth, such as Trisomy 13. No infants were excluded based on clinical acuity. After extensive chart review, 69 infants were eligible for the study.

### 2.2. Comparison and Use of Human Milk Fortifiers

Maternal breast milk (MBM) was fortified according to manufacturer directions. Ingredient and estimated nutrient compositions of fortified preterm human milk were obtained from online nutritional references [16,17]. Table 1 provides a composition comparison for key nutrients and ingredients.

**Table 1:** Comparison of ingrediants and key nutrients using powder and liquid HMF.

24-Calorie-Per-Ounce Fortified Premature Human Milk [16,17]		
Per 100 mL	Powder HMF	Liquid HMF
Protein (g)	2.35	3.2
Iron (mg)	0.46	1.85
Calcium (mg)	138	141
Phosphorus (mg)	78	78
Vitamin D (IU)	119	200
pH	-	4.7
Primary Fortifier Macronutrient Ingredients	nonfat milk, whey protein concentrate, corn syrup solids, medium-chain triglycerides (MCT oil)	water, whey protein isolate hydrolysate (milk), medium chain triglycerides (MCT oil), vegetable oil (soy and high oleic sunflower oils)

-: Information not avaliable.

Enteral feedings are initiated in this NICU within the first one to three days of life with Human Milk (MBM as available or donor milk form the Milk Bank of Austin) at 20 mL/kg/day, trophic feedings are continued for three to five days at the discretion of the attending neonatologist, and then feedings are advanced daily by 20 mL/kg/day with human milk fortification beginning at 80–100 mL/kg/day enteral volume. A protein modular is utilized to improve protein intake to approximately 4 g/kg/day enteral protein once caloric density is 24 kcal/oz. While using the ALHMF, no additional protein modular was utilized. There were no other nutrition differences during the two time periods. Nutrition is managed closely per unit protocol and is very consistent from provider to provider.

According to unit policy, infants receiving the PHMF also received supplementation with a protein modular to provide approximately 4 g protein/kg/day when fed at goal volumes.

Sole use of the ALHMF was initiated on April 1, 2011. Infants receiving the PHMF before this date of fortification change were included in the control group (PHMF, *n* = 46). Infants receiving the ALHMF following this date were included in the study group (ALHMF, *n* = 23). Infants transitioned from the PHMF to the LHMF on the date of fortification change were excluded.

### 2.3. Data Collection

Four investigators familiar with the electronic medical record and NICU terminology obtained all data in a consistent predetermined manner. Collected information was reviewed for accuracy and corrected if an electronic error occurred. All available information on each infant was included in the analysis and is displayed in the tables.

### 2.4. Demographics

Demographic information was collected for all infants including gender, gestational age at birth and discharge, and day of life (DOL) at discharge. Additional clinical outcomes were collected including the presence of bronchopulmonary dysplasia (BPD), retinopathy of prematurity (ROP), intraventricular hemorrhage (IVH), necrotizing enterocolitis (NEC), diaper dermatitis, and death. Treatment requirements were coded similarly if an infant required: oxygen at 36 weeks estimated gestational age (EGA), ROP procedure, IVH shunt, Avastin treatment, Dexamethasone use, and Bicitra use. ROP stage, IVH grade, and number of days of Dexamethasone use were included if available.

### 2.5. Anthropometrics

Infants were weighed daily on a gram scale, and head circumference and length (centimeters) were recorded weekly by nursing staff using a measuring tape. Fenton growth curve percentile rankings [18,19] were electronically plotted for each recorded anthropometric measurement. Weight, head circumference, and length measurements with associated Fenton percentile rankings were taken for infants at birth and at 36 weeks EGA, if available.

### 2.6. Nutrition

Enteral feeding data collected included day of life (DOL) enteral feedings were initiated, DOL full enteral feedings were reached (with a discontinuation of parenteral nutrition support), and the number of times enteral feedings were held (not secondary to preparation for a procedure). Maximum caloric density and number of days on enteral feedings >24 cal/ounce were collected for infants requiring caloric densities higher than the standard 24 cal/ounce to promote adequate growth.

Daily average provision of calories and protein (g) per kg body weight were calculated for infants in each group if they received ≥50% of enteral feedings as fortified MBM during NICU stay. These averages were taken when fortified enteral feedings reached a minimum of 140 mL/kg/day until either daily intake was consistently less than this amount, the infant was changed to unfortified MBM, or the infant received greater than 50% infant formula. Growth and nutrition was evaluated for the groups comparing only growth during the period where the infant received ≥50% of enteral feedings as fortified MBM. An electronic medical system (Intuacare^®^: Omaha, NE, USA) contained protein references for breast milk and specified enteral formulas and caloric density. Nursing staff documented daily intake of breast milk or specified enteral formulas, thus, daily calorie and protein provision per kg of body weight were electronically calculated using the daily-recorded weight. The electronic medical system also calculated the percentages of MBM *vs*. infant formula received according to nursing documentation.

### 2.7. Laboratory Measurements

Maximum creatinine, maximum blood urea nitrogen (BUN) level, maximum base deficit value, maximum calcium level, and lowest carbon dioxide (CO_2_) lab values were collected, if available, after DOL 14 and DOL 30 for all infants. Values were not collected before DOL 14 to eliminate those reflective of parenteral nutrition support and unfortified enteral feedings. Phosphorus and pH were not consistently nor routinely obtained in this patient population and were therefore not collected in this retrospective study.

### 2.8. Data Analysis

Descriptive statistics were displayed for all variables by type of milk (powder *vs*. liquid) given. The Wilcoxon rank sum test was used to compare continuous data between the milks groups. Associations of categorical variables were assessed with the Fisher’s exact test. A *p*-value ≤ 0.05 was considered statistically significant. To assess the difference in growth patterns between infants given powder and infants given liquid, a mixed effects model was used. We included random slopes and intercepts for each subject to capture individual growth pattern as well as fixed effects for group and day and a group day interaction term. A significant interaction of day and group indicates differing growth patterns based on group. Growth Velocity (GV) was calculated using Equation 1 [20].

GV = [1000 × ln(*W*_n_/*W*_1_)]/(*D*_n_ − *D*_1_) (1)

## 3. Results

There were 46 infants in the PHMF group (21 males, 25 females) and 23 infants in the ALHMF group (13 males, 10 females) (*p* = 0.45). Additional baseline characteristics were not statistically significant between the two groups, as shown in Table 2. Enteral feeding data, growth and analyzed lab values are displayed in Table 3. Clinical outcomes are displayed in Table 4. ROP stage, IVH grade, and number of days of Dexamethasone use were not statistically significant and are not included in Table 4.

**Table 2:** Baseline characteristics of the subjects.

Variable	PHMF			ALHMF			*p*-value
	*n*	Mean	SD (±)	*n*	Mean	SD (±)	
**CGA at Birth**	46	29.5	3.0	23	30.3	2.5	0.21
**Birth Weight (g)**	46	1293.7	407.5	23	1437.3	375.6	0.13
**Birth Weight Percentile**	46	31.4	24.7	23	36	26.5	0.82
**Weight at 36 Weeks CGA (g) ^#^**	44	2245.9	450.72	18	2071.2	367.4	0.17
**Weight Percentile at 36 Weeks CGA ^#^**	44	18.6	24.4	18	10.3	13.8	0.22
**HC at Birth (cm)**	46	27.2	3.4	22	27.9	2.1	0.19
**HC Percentile at Birth**	46	29.9	23.1	22	33.6	26.3	0.7
**HC at 36 Weeks CGA (cm) ^#^**	42	32.5	2.6	19	31.9	1.5	0.37
**HC Percentile at 36 Weeks CGA ^#^**	42	38.8	30.7	19	31.4	24.6	0.5
**Length at Birth (cm)**	46	38.6	3.9	21	40.4	2.8	0.07
**Length Percentile at Birth**	46	31.4	24.6	22	32.8	21.9	0.68
**Length at 36 Weeks CGA (cm) ^#^**	42	44.2	3.3	19	43.5	4.6	0.44
**Length Percentile at 36 Weeks CGA ^#^**	42	17.3	22.3	19	21.3	28.1	0.93

^#^ Growth at these time points represents nutrition delivery throughout hospitalization not just breast milk with PHMF and ALHMF.

**Table 3:** Enteral feeding, growth and laboratory data.

Variable	PHMF		ALHMF				*p*-Value
	*N*	Median		*N*	Median		
**Average Daily Provision of Protein per kg Weight**	42	3.9		18	4.3		0.0014
**CO2 Minimum after DOL 14**	33	23		16	18.5		0.002
**CO2 Minimum after DOL 30**	23	25		8	20		0.002
**Growth Velocity (g/kg/day) while on HMF**	46	15.37		21	10.59		<0.0001
**Growth (g/day, while on HMF)**	46	31.27		21	23.66		0.0001
**DOL Enteral Feedings Started**	46	3.0		22	1.1		0.12
**Calcium Maximum**	34	10.3		16	10.45		0.17
**BUN Maximum after DOL 14**	33	18		16	20		0.28
**BUN Maximum after DOL 30**	23	18		8	16		0.91
**Creatinine Maximum**	46	0.92		22	0.9		0.52

**Table 4:** Clinical outcomes.

Variable	PHMF	LHMF	*p*-Value
	*n* (%)	*n* (%)	
NEC	0 (0%)	3 (13%)	0.03
ROP	16 (35%)	3 (13%)	0.09
ROP Procedure	3 (7%)	2 (9%)	1.00
IVH (any)	18 (39%)	4 (17%)	0.10
Dexamethasone Treatment	9 (20%)	1 (5%)	0.15
Bicitra Treatment	0 (0%)	1 (5%)	0.31
Death	0 (0%)	1 (4%)	0.33
Diaper Dermatitis	5 (11%)	4 (18%)	0.46
BPD	9 (20%)	3 (14%)	0.74

### 3.1. Safety and Clinical Outcomes

**Figure 1:**
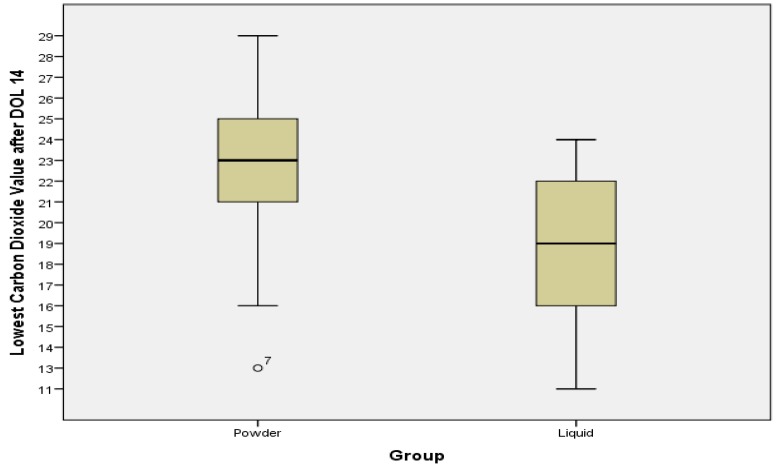
CO2 levels between groups after Day of Life 14. The lowest CO2 levels after DOL 14 were collected from metabolic panels. The mean level in the powder group was 23, the mean level in the liquid group was 18.5. Laboratory clinical reference range 22–32 mmol/L. The difference is statistically significant (*p* = 0.002).

**Figure 2:**
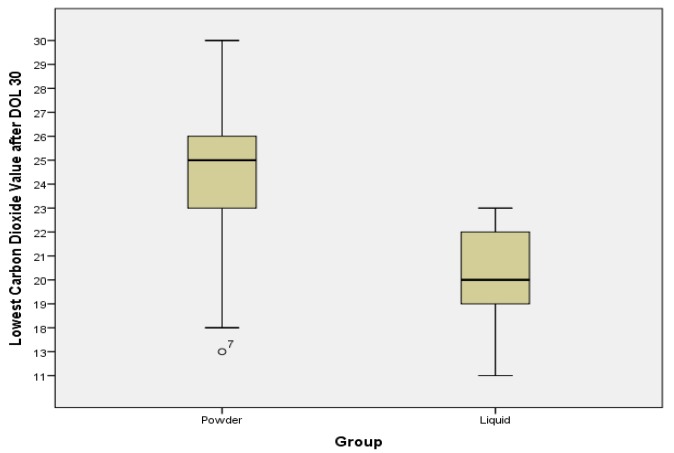
CO2 levels between groups after Day of Life 30. The lowest CO2 levels after DOL 30 were collected from metabolic panels. The mean level in the powder group was 25, the mean level in the liquid group was 20. Laboratory Clinical reference range 22–32 mmol/L. The difference is statistically significant (*p* = 0.002).

Mean lowest CO_2_ lab values (collected while infants were enterally feeding and not acutely ill) were significantly lower in the ALHMF group compared to the PHMF group after both DOL 14 (18.5 *vs*. 23 mmol/L, *p* = 0.002) and DOL 30 (20 *vs*. 25 mmol/L, *p* = 0.002). Lowest CO_2_ lab values after DOL 14 are displayed comparatively in Figure 1. Lowest values after DOL 30 are displayed similarly in Figure 2. Maximum BUN and creatinine levels were similar between the two fortifier groups and were not statistically significant. All other analyzed lab values were not statistically different. All laboratory data in this retrospective study was obtained for clinical purposes regardless of the fortifier group.

Incidence of NEC (a variable we were not powered to evaluate) was significantly higher in the ALHMF group compared to the PHMF group (13% *vs*. 0%, *p* = 0.03).

### 3.2. Enteral Nutrition and Growth

**Figure 3:**
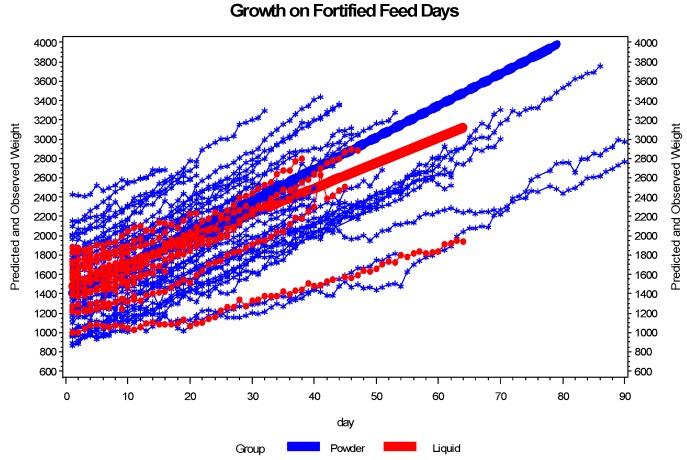
The growth pattern of infants receiving powder differs from the growth pattern of infants receiving liquid on fortified feed days. The plot shows the growth pattern for each infant and the fitted line by group. Based on the plot, infants on powder grow at a faster rate than infants receiving liquid. Evaluation of growth in gm/kg/day for the days infants were fed fortified breast milk, based on the mixed effects model, shows a significant interaction between day and group (*p* = 0.0022). Truncating the analysis at 45 days did not attenuate the results.

Growth was significantly different between the two groups as measured in g/kg/day and is described in Figure 3. Infant growth as measured in g/day from birth to 36 weeks EGA was 23.7 in the PHMF group and 18.8 in the LHMF group (*p* = 0.057). There were no statistically significant differences in the length of time to full feedings or the number of times that feedings were held that could account for the difference in growth rates between the two groups.

Daily average protein/kg/day provision was higher in the ALHMF group compared to the PHMF group (4.3 *vs*. 3.9 g, *p* = 0.0014). Mean enteral calorie provisions in the ALHMF group were higher than in the PHMF group, 117.3 kcal/kg/day in the PHMF group as compared to 128.7 kcal/kg/day for infants in the ALHMF group (*p* = 0.057). A higher proportion of infants in the ALHMF group required increased caloric density of feedings >24 cal/ounce as compared to infants in the PHMF group, (48% *vs*. 26%, *p* = 0.10). While this did not reach a statistical difference, clinically this was notable.

## 4. Discussion

To our knowledge, we are the first study to date to report our clinical findings of increased complications with the use of ALHMF in a Level IIIc clinical setting. In our retrospective analysis of acidosis, growth, and clinical outcomes in NICU infants fed with human milk fortified with LHMF and PHMF we found significant acidosis and poor growth in the infants receiving LHMF. These findings were very consistent with our clinical impressions during our clinical use of the LHMF. We were also surprised to see increased NEC in the ALHMF group. Although we were not powered as a primary outcome to evaluate NEC, we strongly encourage cautious further evaluation of the product in the clinical setting with regards to this serious outcome.

A key difference in the ALHMF as compared to the PHMF is the acidification process required for sterilization. This difference is likely to explain the increased complications seen in the ALHMF group. The preterm infant’s inability to buffer this acid load likely led to an increase in clinical complications including acidosis, poor growth, and, possibly, NEC.

### 4.1. Acidosis

There was a higher incidence of clinically significant metabolic acidosis in the ALHMF group, with one infant requiring treatment with Bicitra. No infants in the PHMF group required Bicitra treatment, even with twice as many patients in this group. Premature infants are susceptible to metabolic acidosis [21] and renal tubular acidosis. However, these imbalances of acid base status should begin to normalize after the first weeks of extrauterine life [21]. Considering similar baseline characteristics, we hypothesize additional enteral acid load was a potential contributor to this increased incidence of metabolic acidosis in the LHMF group.

Premature infants are at risk for developing metabolic acidosis secondary to immature metabolic processes, a lower renal capacity to adequately excrete acid, and higher urinary losses of bicarbonate [2,4,22]. Quantity of protein may affect metabolic processes; however the median daily average protein provisions for each fortifier group were within the currently recommended ranges [1]. No clearly defined amount for maximum protein provision exists, however, it is suggested that intakes greater than 6 g/kg/day are poorly tolerated [2]. Maximum daily average protein provisions for both groups were below this level. Another reference states that protein provisions greater than 5 g/kg/day may cause azotemia [1], but each group had intakes below this value, and maximum BUN and creatinine levels were not different in the two fortifier groups. Having increased protein intake in the PHMF group as well as the ALHMF group helps to illuminate that increased protein content in the ALHMF was not likely the cause of the adverse outcomes.

We question if the acidification sterilization process of the ALHMF may contribute to this acidosis in some fragile premature infants. Our patient population included in this study was not limited by respiratory acuity, as was the population in the Moya *et al*. paper [23]. We hypothesize that our more inclusive population of both healthy infants and more fragile infants who may have less respiratory stability decreases their capability to buffer the acid load provided in the ALHMF resulting in clinical acidosis in some cases requiring medical therapy. It may be unwise in a fragile preterm infant population to minimize the clinical significance of the metabolic acidosis noted in the ALHMF groups in our study, the study conducted by Moya *et al*. [24], who reported that infants fed the ALHMF had significantly lower pH (at day six), bicarbonate (at day six and 14), and CO_2_ (at day 14 and 28), and significantly higher chloride (at day 14 and 28). Additionally, in an abstract evaluating 100 infants, 50 fed with ALHMF and 50 fed with PHMF, published by Cibulskis *et al*., from Saint Louis University at the 2013 AAP_NCE, similar metabolic acidosis is described in this patient population (54% ALHMF *vs*. 10% PHMF, *p* = 0.0001) [25]. As reported in their abstract, this grouptreated the acidosis as if it were clinically significant, discontinuing ALHMF on 21/50 patients due to a clinical diagnosis of acidosis [25].

### 4.2. Enteral Nutrition and Growth

Infants in the PHMF group received a mean daily calorie intake of 117.3 kcal/kg/day as compared to infants in the ALHMF group who received a mean calorie intake of 128.7 kcal/kg/day. Infants in the ALHMF group also received a median of 0.4 g protein/kg/day more than the infants in the PHMF group. Despite higher protein and calorie provisions in the LHMF group, growth during the HMF period was slower between the two groups as evaluated by several methods: in a mixed effects model evaluated in g/kg/day (*p* = 0.002), in g/day (*p* = 0.0001), and by growth velocity in g/kg/day (*p* < 0.0001). Noted also, is that ALHMF infants experienced an additional decrease of 10 growth curve percentiles for weight from birth to 36 weeks EGA when compared to infants in the PHMF group (growth at 36 weeks is representative of nutrition delivery that is not limited to the period evaluated on PHMF and ALHMF). As Dexamethasone use inhibits growth in premature infants [4], we further note that fewer infants in the ALHMF group (5%) compared to the PHMF group (20%) required this drug for clinical treatment (*p* = 0.15).

Maintaining appropriate growth in this patient population was a high priority, so infants with suboptimal growth were fed increased caloric density feedings above 24 cal/oz. Though not statistically significant, a higher proportion of infants in the ALHMF group (48%) required caloric densities greater than 24 cal/ounce when compared to the PHMF group (26%). Had those 48% of infants in the ALHMF group not been prescribed increased caloric densities due to clinical observations of poor growth differences in growth throughout the hospitalization would likely have been larger between the PHMF groups and ALHMF groups. The statistical significance in infant growth as noted in g/kg/day is seen in spite of the high priority our unit takes in maintaining optimal growth and the subsequent aggressive adjustment of caloric density to achieve desired results. This was ultimately the reason 26% of infants receiving ALHMF were transitioned to receive the PHMF once the ALHMF use was discontinued in the NICU.

Not only are these growth effects consistent with the findings of Moya *et al*., they raise further questions [24]. Moya *et al*., reported no significant differences in rate of weight gain or head circumference growth between infants fed this same ALHMF and infants fed a PHMF, even though the human milk fortified with ALHMF contained 23% more protein (3.2 *vs*. 2.6 g protein/100 mL fortified preterm human milk) [24]. In our study, even though we compensated for the difference in protein content so that protein intake was similar, there was still poorer growth in infants fed ALHMF.

At least part of the inability of the additional protein to improve growth may be due to the acidosis noted above. It is well-known that infants with metabolic acidosis hyperventilate, as the expiration of CO_2_ drives the elimination of H^+^ ions through the bicarbonate buffering system. What is less well recognized is that protein catabolism can also be utilized to decrease acidity by the elimination of H^+^ ions through the urinary excretion of NH_4_^+^. Acidosis reduces protein synthesis in rats [26] and leads to protein catabolism in humans [27].

### 4.3. NEC

No infants in PHMF group developed NEC compared to 13% in the ALHMF group. Reasons for these occurrences remain unclear, as similar prevention strategies were followed for each group. Previous implementation of aggressive nutrition practices in our unit demonstrated improved feeding tolerance and clinical outcomes, with no increased incidence of NEC [12]. These nutrition practices remained unchanged during the study period, and no additional clinical practices were implemented concurrent with the change in human milk fortification. Slow rate of enteral feeding advancement remained consistent between both groups, as evidenced by no statistically significant differences in length to full enteral feedings. No changes in brand or caloric density of premature infant formula were made, and infant formula was utilized equally in both groups if no MBM was available. As no additional practice changes were implemented during this study period, we can neither confirm nor exclude use of the ALHMF as a contributor to these occurrences of NEC. Although this study was not powered to detect NEC based on historical incidence in our unit with rates over the last five years ranging from 2% to 5% from our Vermont Oxford Network data, one should consider that significant differences with small sample sizes may either reflect coincidental effects due to sample size, or may be due to a real difference that is unexpectedly large.

### 4.4. Metabolic Acidosis

Literature suggests that premature infant formulas contain a high renal acid load, though human milk contains less [2,21]. Research has additionally demonstrated that the composition of infant formulas may affect the urinary pH and nutrient excretion of premature infants [21,22]. It is further proposed that high renal acid loads contribute to maximum renal acid stimulation (urine pH < 5.4) [28] in premature infants with immature renal function. Previous research studies have demonstrated that infants with metabolic acidosis or maximum renal acid stimulation exhibit decreased growth [28,29]. This may also result in an increase in urinary sodium excretion [24,29] and a decrease in nitrogen assimilation [30]. Blood sampling for acid-base indicators may not be significantly abnormal in the presence of maximum renal acid excretion [22,28]. However, CO_2_ values may trend low [28], which was clearly observed among infants in the ALHMF group (*p* = 0.002).

### 4.5. Summary

Our results showing increased acidosis in the ALHMF group raise further concerns with use of the ALHMF, as infants with metabolic acidosis may experience altered nutrient metabolism [28,31] and decreased bone mineralization [32], leading to poor growth and osteopenia of prematurity.

Poor growth in the ALHMF group may also be attributed to changes to the nutrient content of the milk caused by acidification as described by Erickson *et al*. [23]. This group reported significant changes in acidified breast milk, including decreased total protein content, lipase activity, and free fatty acids [23]. The nutritional changes in the composition of acidified breast milk documented by Erickson *in vitro* may have led to the *in vivo* growth deficiencies noted in our ALHMF population [23].

## 5. Strengths and Limitations

### 5.1. Strengths

This study is the first to quantify results of use of ALHMF in a Level IIIc NICU setting. We are uniquely situated to evaluate outcomes of our use of ALHMF in our patient population for several important reasons. First, we initiated utilization of this product on all infants at one time. There was no possibility of crossover product use to decrease the validity of the data. Additionally, we used this product on all infants who would be eligible to receive fortified human milk, as would be expected in a clinical NICU practice. This makes our data very relevant and applicable to clinical NICU settings.

Second, our clinical management of nutrition in this patient population has been published and remains very successful with excellent growth and low baseline rates of NEC. Not only do we manage nutrition care of this population very closely, but we also have a defined protocol in place so that infants (except for fortification method) receive the same nutrition interventions over time regardless of which group, PHMF or ALHMF, they received.

Additionally, our nutrition management with additional protein added to the PHMF group makes the comparison of the two groups more relevant by giving them a more similar nutrient intake at baseline than a comparison of ALHMF and PHMF alone which compares a large difference in delivered protein.

Finally, we have a very detailed nutrition documentation medical record system, Intuacare. This system allows for easy retrieval of detailed nutrition information including daily percentages of breast milk, daily caloric intake, and daily protein intake in g/kg/day. This allows for minimal reporting error in a retrospective study, such as this, and provides an excellent historical representation of each infant’s delivered nutrition.

### 5.2. Limitations

This retrospective review of a clinical trial of a commercially available acidified liquid human milk fortifier has several limitations including the retrospective nature of the study, and a modest sample size, which limits the power of some data points. These limitations were partially reduced by our reliance on electronic documentation for data collection and analysis. All medical documentation remains variable between individuals and we cannot quantify unrecorded data, but the system utilized allowed for complete assessment of all recorded data on each research subject. As with any study evaluating growth, head circumference and length measurements are also variable as length boards were not used and measuring tape placement may vary between nursing staff. Some subjects were discharged prior to 36 weeks EGA, therefore, anthropometric measurements at 36 weeks EGA were not available. Likewise, lab values were also unavailable for these infants and could not be included in data analysis.

Alterations in human milk composition are continuous, so calculated nutrient compositions of fortified human milk may only serve as general estimations for our nutrient comparisons. Standard NICU nutrition practices are followed as consistently as possible, however feeding advancement may remain variable according to infant clinical status. Furthermore, the proportion of feedings as human milk or formula remained variable among each infant. In an ideal study, all enrolled infants would receive human milk only.

Though the incidence of NEC was statistically significant, it was not powered as a primary outcome for this study. We also suspect that diaper dermatitis was under-recorded during this study period, as our clinical experience suggests that diaper dermatitis is infrequently documented in the electronic medical record even when infants experience more serious medical complications. However, perceived worsening skin breakdown in our unit while using the ALHMF prompted development of a unit list of infants with diaper dermatitis. Unfortunately, not all of the infants recorded on the unit list had diaper dermatitis electronically documented as a medical problem. As there was no way to quantify these cases, these select infants were not coded positively for diaper dermatitis in this study. Therefore, our data analysis remains limited.

## 6. Conclusions

Use of the new ALHMF resulted in an increase in clinical complications and a decrease in growth as measured in both g/day and g/kg/day. To our knowledge, this is one of the first studies assessing use of the new ALHMF within a high acuity NICU without excluding infants with significant respiratory disease or low five-min APGAR scores. Further research with the ALHMF is needed to compare infant tolerance and outcomes among infants with a variety of gestational ages, weights, and increased clinical acuity.
